# Female large odorous frogs (*Odorrana graminea*) prefer males with higher nonlinear vocal components

**DOI:** 10.1002/ece3.8573

**Published:** 2022-02-10

**Authors:** Pan Chen, Jinmei Wang, Junqi Miao, Hao Dong, Jiahui Bao, Yatao Wu, Fang Zhang

**Affiliations:** ^1^ 12514 Anhui Provincial Key Laboratory of the Conservation and Exploitation of Biological Resources College of Life Sciences Anhui Normal University Wuhu Anhui P. R. China; ^2^ 12514 School of Ecology and Environment Anhui Normal University Wuhu Anhui P. R. China

**Keywords:** acoustic communication, large odorous frog, mate choice, nonlinear phenomenon, phonotaxis

## Abstract

In anurans, the complexity of courtship calls may affect female mate choice. The current study suggests that nonlinear phenomena (NLP) components can contribute to increasing complexity in courtship calls and attracting female attention. The results of a recent study showed that calls of large odorous frog (*Odorrana graminea*) contained NLP components. However, whether the nonlinear components of courtship calls in *O. graminea* improve male attractiveness remains unknown. We hypothesized that female *O. graminea* would prefer males producing calls with a higher proportion of NLP components (P‐NLP‐C). To test this hypothesis, we recorded the advertisement calls of 28 males and confirmed that the P‐NLP‐C was significantly positively related to body size. We also measured the body size of natural amplectant males and non‐amplectant males in the field and found that amplectant males had larger body sizes than non‐amplectant males, and the results of two‐choice amplexus experiments similarly revealed a female preference for males with larger body sizes. Additionally, phonotaxis experiments also revealed that females preferred male calls with a high P‐NLP‐C. The results suggest that a higher P‐NLP‐C in calls can enhance male attractiveness, and the P‐NLP‐C may provide key information about male body conditions for female *O. graminea*. Our study provides a new insight for better understanding the role of NLP in anuran mate selection.

## Graphical abstract

The results suggest that a higher proportion of nonlinear phenomena components (P‐NLP‐C) in calls can enhance male attractiveness, and the P‐NLP‐C may provide key information about male body conditions for female *Odorrana graminea*. This study provides a new insight for better understanding the role of NLP in anuran mate selection.

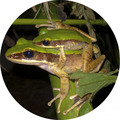

## 1 INTRODUCTION

Sound communication plays a vital role in female mate choice, especially in anuran species (Narins et al., 2006; Zhang et al., 2015). The characteristics of male courtship call, particularly the complexity of the call, are important factors to be considered by gravid females when choosing a mate (Cui et al., 2016; Ryan et al., 2019). Females attend to the calls to assess males and have a stronger preference for complex calls over simple calls (Gridi‐Papp et al., 2006). The acoustic complexity is often associated with variations in the spectrotemporal characteristics (e.g., frequency, duration, and the number of syllables) of the vocal signals (Fee et al., 1998; Márquez, 1995; Ryan, 1983). In some species producing nonlinear calls, improving the content of nonlinear phenomena (NLP) is an important way to increase the call complexity (Fitch et al., 2002; Rice et al., 2011; Wu et al., 2021).

Nonlinear acoustic phenomena generally have complex structures (Rebout et al., 2020). Two asymmetrical vocal folds, with unequal masses or lengths, induce nonlinear phenomena (NLP), which include four acoustic characteristics, that is, frequency jumps, subharmonics, deterministic chaos, and biphonations (Fitch et al., 2002). NLP, as distinctive structural features of acoustic signals, are ubiquitous among the acoustic signals of vertebrates (Digby et al., 2014; Feng, Riede, et al., 2009; Fitch et al., 2002; Rice et al., 2011; Zhang et al., 2017). The occurrence and strength of NLP in calls not only increase individual vocal distinctiveness but also attract more attention from receivers in individual interactions, which was widely confirmed in fish and mammals (Fitch et al., 2002; Volodina et al., 2006; Wilden et al., 1998). Several studies have focused on the general function of NLP, for example, in alarm signals (Blumstein & Recapet, 2009; Townsend & Manser, 2011) and to convey information about direction and identity (Digby et al., 2014; Kaplan et al., 2018), but few have focused on mating choice, especially in frogs (Wu et al., 2021).

The large odorous frog (*Odorrana graminea*) can produce calls that contain four NLP components (subharmonics, deterministic chaos, frequency jumps, or biphonations), and 84.6% of the vocalizations contain one or more nonlinear components (Zhang et al., 2021). *O. graminea* is an arboreal species inhabiting areas around loud streams and waterfalls in select regions of China (Chen, 1991). Similar to that of a sympatric species, *Odorrana tormota*, the calls of *O. graminea* consist of ultrasonic and NLP components (Shen et al., 2011; Zhang et al., 2021). In *O. tormota*, smaller males with higher call frequencies had greater mating success than larger rivals, as their calls were more conspicuous in the species’ habitat with intense but predominantly low‐frequency stream noise (Zhang et al., 2020). Whether the mode of mate choice in *O. graminea* is similar to that in *O. tormota* remains unknown. Furthermore, since NLP components can improve the complexity of courtship calls, knowledge about the roles of NLP levels in attracting mates is essential for understanding mate choice as a social function, but studies on the roles of NLP levels in mate attraction have been conducted in only one anuran species (*O. tormota*) that inhabits subtropical stream areas (Wu et al., 2021). Therefore, it is unclear whether or how the nonlinear components of courtship calls improve male *O. graminea* attractiveness and whether females of *O. graminea* prefer males with a higher proportion of NLP components (P‐NLP‐C). To address these issues, we investigated (1) the mode of mate choice in *O. graminea* and (2) the effects of P‐NLP‐C on mate choice.

## 2 METHODS

### 2.1 Study site and subjects

Field observations and laboratory experiments involving *O. graminea* were conducted at Fu Creek in Huangshan, Anhui Province, China (118°08′44.89″E, 30°05′01.61″N, elevation: 600 m asl). Fieldwork at the location over the past seven years has revealed that the peak month for breeding activity in *O. graminea* is May. As mate choice may vary with different periods in the breeding season, field and laboratory studies were carried out during peak breeding season, from May 2 to May 28 in 2019 and 2020, to minimize sampling bias. Each night, we captured as many amplexed pairs as possible during the peak reproductive hours (between 19:30 and 22:30; Figure 1). All captured individuals were placed in containers with water and stones in time and then brought back to the test chamber. The local nightly temperature and humidity during the study period ranged from 19 to 24°C and 90% to 96%, respectively. The test chamber was located near the sampling site and maintained a similar temperature, humidity, and dark light environment. Each individual was in the test chamber for no more than three days and released back to the sampling site immediately after testing. To avoid repeated capture, each frog was given a finger cutting mark before returning to its habitat. All the behavioral observations conducted in this study were performed in accordance with the current laws of the China and the Animal Care and Use Committee at the Anhui Normal University (Permit # 00111).

**FIGURE 1:**
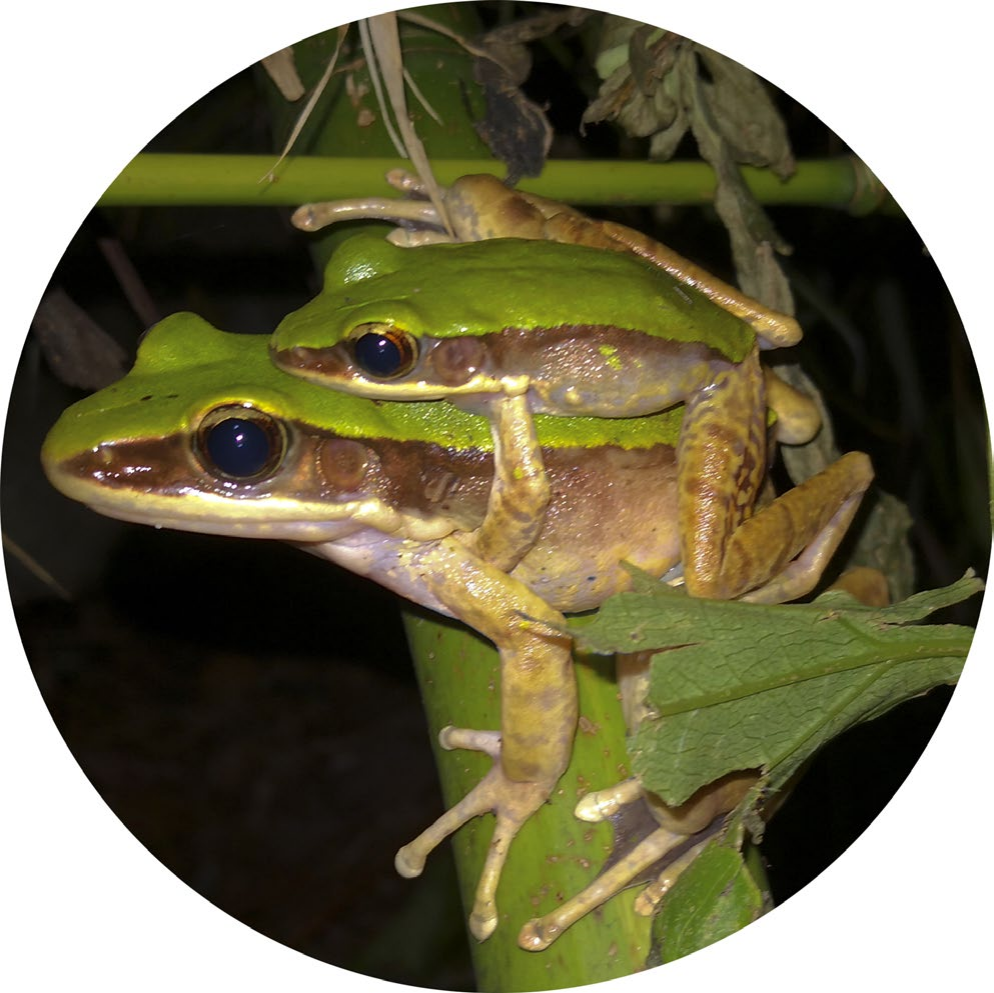
An amplexed pair of Odorrana graminea in the field. Photo by Jinmei Wang

### 2.2 Field sampling

We captured 30 amplexed pairs that were found on top of boulders in the middle of the mountain stream and in bamboo groves next to small waterfalls in the stream. Three to six solo males (i.e., non‐amplectant) in the vicinity (< 0.5 m) of an amplexed pair were randomly captured and housed in separate terrariums (22 × 17 × 15 cm). Using a caliper (Spi2000 Wiha, Germany) with an accuracy of ±0.1 mm, we measured the snout‐vent lengths (SVLs) of the frogs. The SVL data of the amplectant males were later compared to those of solo males. The number of amplexed pairs we spotted and captured each night varied from 0 to 5. No amplexed pair was found in the field when the ambient temperature was below ~15 °C. A total of 123 solo males were captured, and their characteristics were compared with those of amplexed males in the field (Table 1).

**TABLE 1:** The SVLs of amplectant and non‐amplectant males from nightly field observation. Shown are the mean ± SD

Night #	Mean SVL of amplectant males (mm)	Mean SVL of non‐amplectant males (mm)
1#	50.45 ± 2.77 (*N* = 4)	48.52 ± 2.48 (*N* = 16)
2#	50.52 ± 2.52 (*N* = 5)	49.85 ± 2.19 (*N* = 21)
3#	49.90 ± 3.10 (*N* = 5)	48.82 ± 2.18 (*N* = 19)
4#	50.75 ± 2.10 (*N* = 4)	49.54 ± 2.20 (*N* = 16)
5#	50.15 ± 1.20 (*N* = 2)	49.07 ± 2.23 (*N* = 9)
6#	51.30 ± 0.57 (*N* = 2)	48.79 ± 3.20 (*N* = 11)
7#	51.15 ± 1.20 (*N* = 2)	49.93 ± 2.41 (*N* = 9)
8#	51.70 ± 0.57 (*N* = 2)	48.30 ± 3.65 (*N* = 8)
9#	51.25 ± 1.48 (*N* = 2)	47.36 ± 2.37 (*N* = 8)
10#	51.20 ± 0.42 (*N* = 2)	49.83 ± 2.12 (*N* = 6)
Total	50.74 ± 2.01 (*N* = 30)	49.07 ± 2.48 (*N* = 123)

### 2.3 Audio recording and call analysis

To quantitatively analyze the relationship between male body size and the P‐NLP‐Cs in vocalizations, we recorded the vocalizations of 28 actively calling males (not in amplexus) for three evenings under similar ambient conditions (temperature: 20 ~ 22°C, humidity: 92% ~ 96%, ambient noise: 70 ~ 78 dB SPL peak) in the field and measured their SVLs. Male calls were recorded by using a digital audio recorder (Sound Devices 702, Sound Devices, WI, USA, frequency range: 10 Hz ~ 96 kHz) with a sampling rate of 96 kHz and 16‐bit accuracy and a miniature omnidirectional condenser microphone with a flat frequency response over 20 ~ 20,000 Hz (AKG model C417, AKG Acoustics, Vienna, Austria) (Zhang et al., 2015). For each male, we recorded at least 8 calls.

**FIGURE 2:**
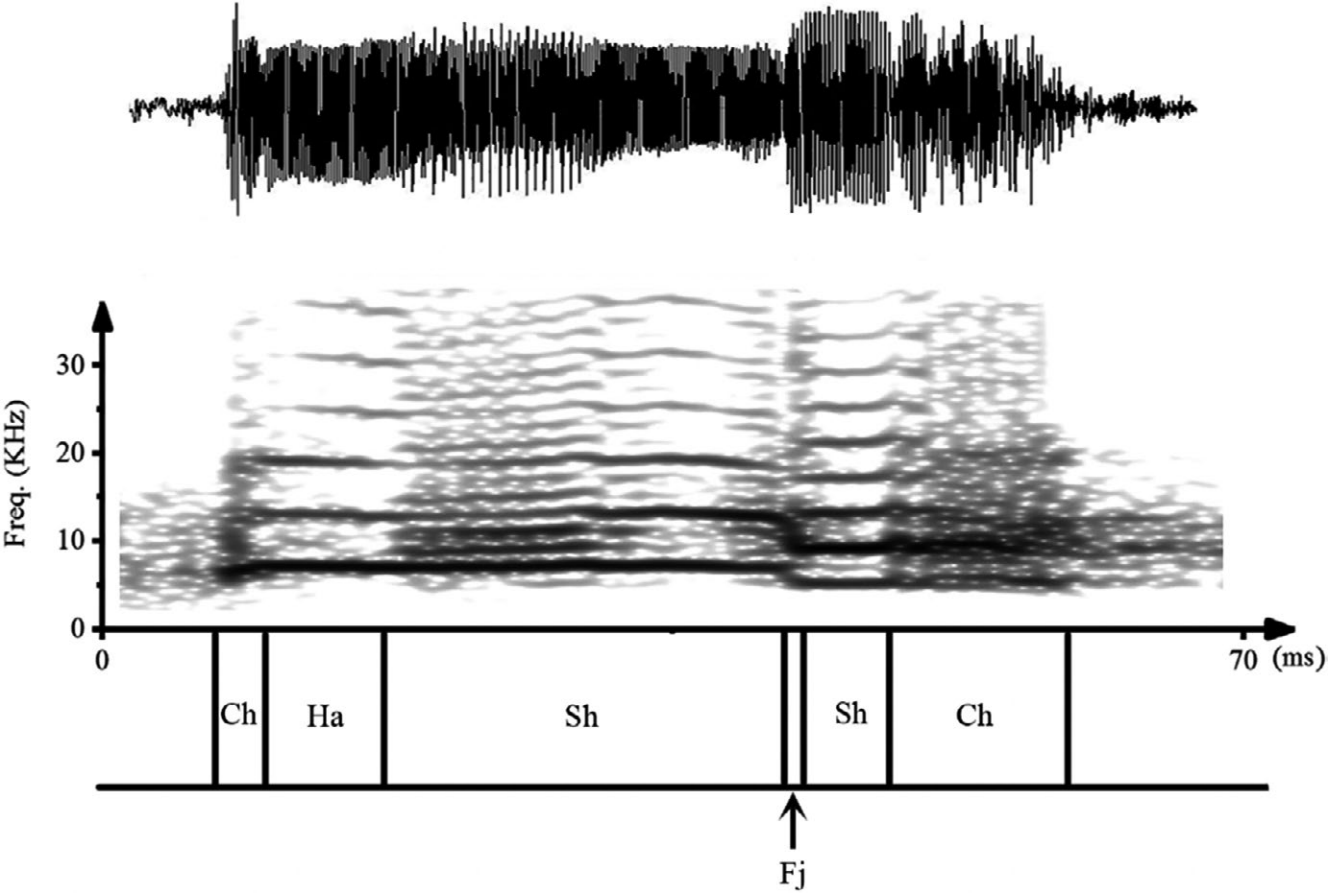
Temporal segmentation of calls. Shown here is the waveform (top trace) and spectrogram (bottom trace) of a call of male Odorrana graminea. The call was segmented into segments containing harmonic (Ha), subharmonics (Sh), deterministic chaos (Ch), and frequency jump (Fj). This particular call does not show biphonation segments

Calls were initially analyzed with SELENA, a custom‐designed program (Feng, Riede, et al., 2009), to produce narrow‐band spectrograms and determine the call durations. Next, calls comprising multiple notes, the duration of individual notes, the duration of signal breaks, and the overall call duration were measured. Then, different temporal segments of each call were identified with PRAAT based on visual inspection of the narrow‐band spectrogram (Figure 2; Boersma & Weenink, 2007). After the call segments were finished, the time of occurrence of each segment was noted. In addition, the durations of the various NLP segments (i.e., subharmonics, deterministic chaos, frequency jumps, biphonations) were measured. Finally, these durations were calculated as percentages of the total call duration, and the average NLP percentage for each male frog was calculated. The fundamental frequency (*f*_0_) was tracked for each harmonic segment using the “pitch tracking” mode in PRAAT, with a 1‐ms interval.

### 2.4 Amplexus experiments

To determine the male body size preferred by females, we performed two different two‐choice amplexus experiments. The behaviors of the males and females were recorded by a camcorder (Sony model HF M40). In the first two‐choice experiment (Experiment A), we paired one gravid female with two randomly captured males with varying body sizes in a test terrarium (38 × 38 × 28 cm) for up to 15 min. We placed the female in the center of the test terrarium and the two males equidistant from the female. If the pairing did not result in amplexus within 15 min, namely, the female rejected both males, we substituted two new males and repeated the experiment (at most one repeat). Upon amplexus, we separated the pair and measured the SVLs of the amplectant male and non‐amplectant male. In total, 27 out of 30 experiments resulted in amplexus.

In the second two‐choice experiment (Experiment B), a female was paired with two males that had been previously paired with a different female. The goal was to determine whether a particular male phenotype was preferred by different females. To execute the second two‐choice experiment, we removed the female previously paired (in Experiment A) from the test terrarium and allowed the males to remain and rest in the terrarium for at least 30 minutes. Then, we placed another female into the terrarium and began the second two‐choice experiment (Experiment B). All individuals in each experiment were randomly assigned and never tested more than once in the same experiment.

### 2.5 Phonotaxis experiments

**FIGURE 3:**
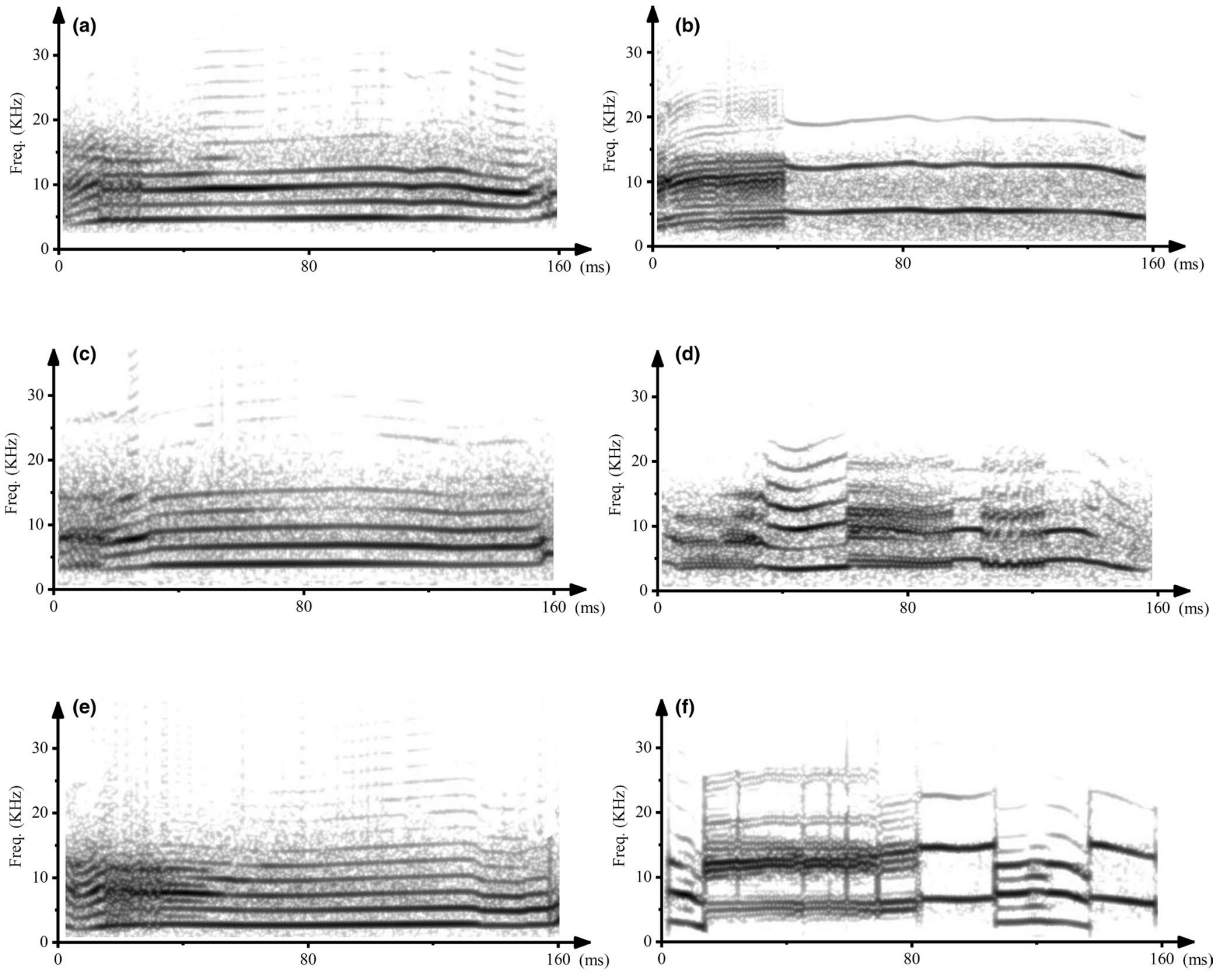
Spectrogram of stimuli pairs. (a) (NLP: 10%; *f*_0_: 2600 Hz) and (b) (NLP: 13%; *f*_0_: 7500 Hz) are a stimuli pair for phonotaxis test 1. (c) (NLP: 10%; *f*_0_: 3800 Hz) and (d) (NLP: 50%; *f*_0_: 3900 Hz) are a stimuli pair for phonotaxis test 2. (e) (NLP: 20%; *f*_0_: 4900 Hz) and (f) (NLP: 75%; *f*_0_: 5000 Hz) are a stimuli pair for phonotaxis test 3

Three acoustic‐stimulus pairs were synthesized using Cool Edit Pro 2.1 software based on the advertisement calls of different males that have been shown to attract females. We first chose some typical calls that fits the criteria (specific frequency or P‐NLP‐C), then synthesized stimulus from different call components in a certain ratio, and finally standardized some parameters including the call duration and amplitude. The characteristics of stimulus were based on the *f*_0_ and P‐NLP‐C of the calls observed in this and previous studies (Zhang et al., 2021). In phonotaxis test 1, the goal was to test the effect of frequency variation on female choice while the P‐NLP‐C at a constant low level. The *f*_0_ of the two stimulus were 2600 Hz, representing low‐frequency calls (indicated by L *f*_0_), and 7500 Hz, representing high‐frequency calls (indicated by H *f*_0_), while the differences in the temporal and P‐NLP‐C characteristics were no more than 20 ms and 3%, respectively (Figure 3a,b). In phonotaxis test 2, the goal was to test the effect of P‐NLP‐C variation on female choice while the *f*_0_ at a constant normal level. The P‐NLP‐C in the two stimulus were 10% for calls with a low P‐NLP‐C (indicated by L1‐NLP) and 50% for calls with a high P‐NLP‐C (indicated by H1‐NLP), while the differences in the temporal and *f*_0_ characteristics were no more than 20 ms and 100 Hz, respectively (Figure 3c,d). In phonotaxis test 3, the goal was to test the consistent of female choice for the P‐NLP‐C level of calls compared to test 2 while adjusting the P‐NLP‐C and *f*_0_ in stimulus. The P‐NLP‐C in the two stimulus were 20%, representing calls with a low P‐NLP‐C (indicated by L2‐NLP), and 75%, representing calls with a high P‐NLP‐C (indicated by H2‐NLP), while the differences in the temporal and *f*_0_ (high level) characteristics were no more than 20 ms and 100 Hz, respectively (Figure 3e,f). Stimulus pairs were constructed as follows: (1) L *f*_0_ versus H *f*_0_, (2) L1‐NLP versus H1‐NLP, and (3) L2‐NLP versus H2‐NLP.

We performed the phonotaxis tests in a hemi‐anechoic chamber (L × W × H: 200 × 120 × 150 cm) in which the walls and ceiling were covered by sound‐absorbing cotton (Figure 7a). Moreover, we covered the floor and loudspeakers (Altec Lansing Orbit iM227) with branches and grass and misted water into the air to simulate humidity. For each test, we placed a female under an inverted, acoustically transparent experimental box (27 × 17 × 15 cm) on the floor of the arena at a centrally located release location. We moved this box with a string from outside the chamber to grant the female access to the entire arena, which was illuminated by infrared light. During the tests, the behaviors of females were recorded with a camcorder (Sony model HF40) located outside the chamber.

We broadcasted acoustic stimuli every 15 s (2 stimulus calls per min per loudspeaker, simulating the natural call rate) using loudspeakers on the floor of the chamber. To avoid the potential side biases, we randomized the loudspeaker assignments for each stimulus pair (Zhao et al., 2017). The distance between each loudspeaker and the release location was 100 cm, and both loudspeakers were balanced to provide 80 ± 2 dB SPL at the release location. At the start of the experiment, females were stimulated with a 1‐min natural chorus recording using two loudspeakers. Then, we broadcasted the stimuli for approximately 1 minbefore lifting the experimental box. A choice was confirmed when a female frog approached within 20 cm of a loudspeaker without simply following the wall. To determine whether a particular call characteristic was consistently chosen by the same female, we performed the second phonotaxis experiments using the same females with different positions of the designated target loudspeakers (females were subjected to a rest period of at least 2 h prior to the next test). Next, the time to leave (the time from removing the box until the female exited the release location) and the time to choice (the time from exiting the release location to reaching the choice boundary) were recorded in all experiments, and the averaged phonotaxis time from the two trials was considered the final result. If a female did not approach any loudspeaker within 10 min, we reran the test after a 30‐minute rest period. If the female still failed to approach a speaker, no choice was recorded (Zhu et al., 2017). Six females were found to be about to oviposit before the experiment and released back to their natural habitat in time. In total, 24 gravid females were used in the phonotaxis tests. The order of test 1 and test 2 was random, but test 3 was performed after test 2. The order of all individual in each experiment was random.

### 2.6 Statistics

All statistical analyses were conducted using SPSS 24.0 (IBM SPSS Inc). For all pooled data, we performed the Shapiro–Wilk test and Levene test to determine whether the data were normally distributed and exhibited homogeneous variance, respectively. For data that met these criteria, we performed a parametric test; otherwise, a nonparametric test was used. The Mann–Whitney *U* test was used to analyze differences in body length parameters between amplectant males and solo males in the field, while the paired *t* test was performed to analyze differences in body lengths between amplectant males and non‐amplectant males in the amplexus experiment. Linear‐regression analysis was used to analyze body length correlations between male and female frogs in the amplexus test. In addition, this analysis was also performed to analyze correlations of male body length with the proportions of NLP and the fundamental frequency in male vocalizations. The binomial test was used to evaluate the phonotaxis data for females responding to male calls with different *f*_0_ and P‐NLP‐C. The Mann–Whitney *U* test was used to compare female phonotaxis time between different stimulus pairs in three phonotaxis experiments.

## 3 RESULTS

### 3.1 Female mate choice in the field

**FIGURE 4:**
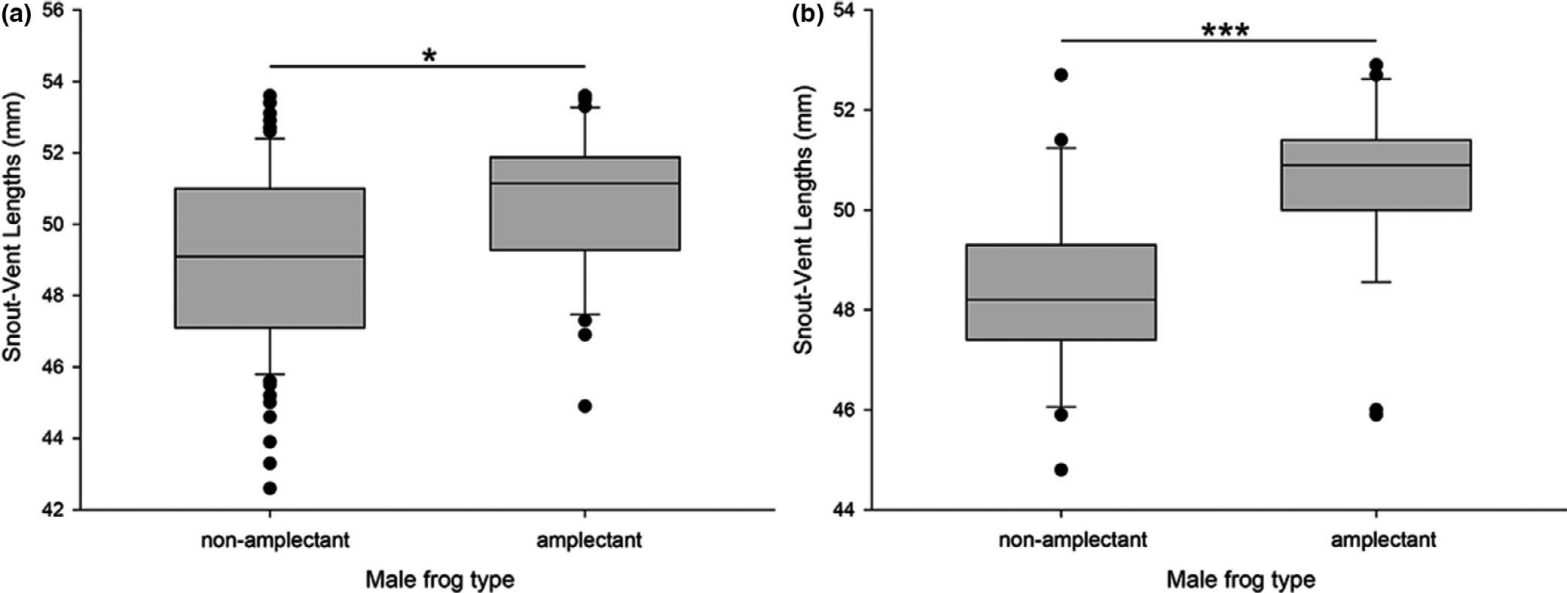
Differences of snout‐vent lengths (SVLs) of amplectant males and non‐amplectant males. (a) Measurements from males derived from field observations (*N* = 30 for amplectant males, *N* = 123 for solo males, Mann–Whitney U test, *p < .05). (b) Measurements from males used in Experiment A performed in the laboratory (*N* = 27 for amplectant males, *N* = 27 for non‐amplectant males, paired *t* test, ***p < .001)

**FIGURE 5:**
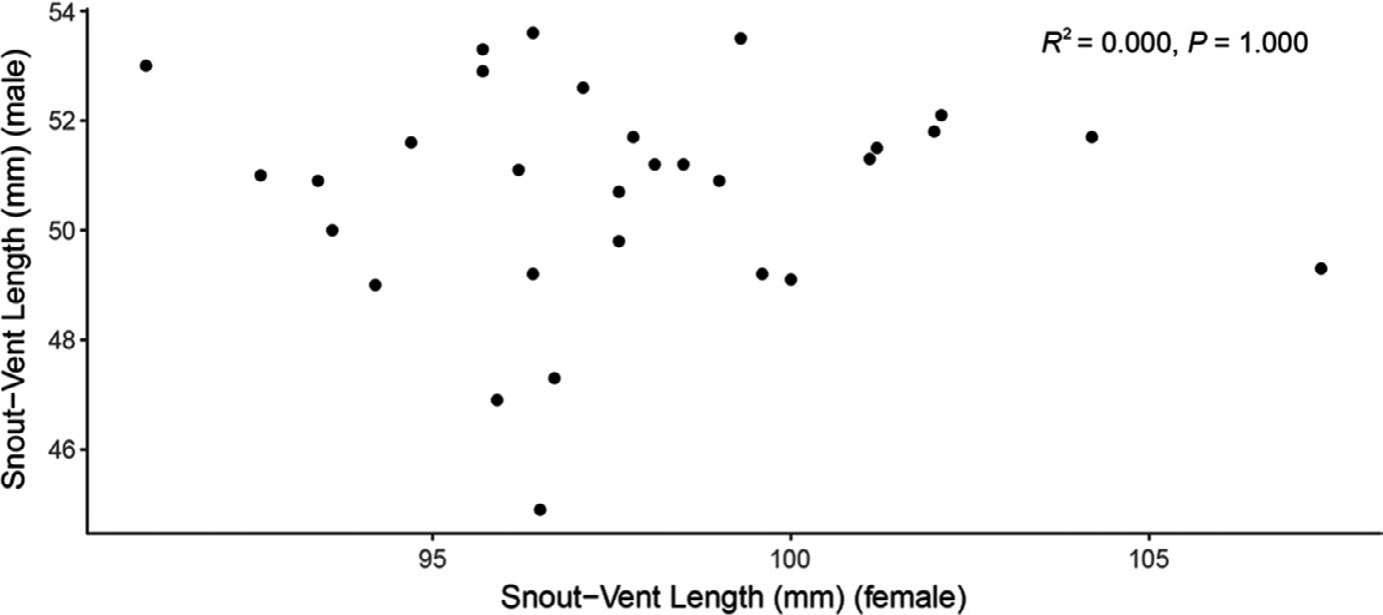
Correlation of the snout‐vent length (SVL) between amplectant males and amplectant females (*N* = 30, linear‐regression analysis, *p* > .05)

The field observations revealed that on average, amplectant males had a larger SVL (mean SVL ± SD = 50.74 ± 2.01 mm, *N* = 30) than non‐amplectant males (mean SVL ± SD = 49.07 ± 2.48 mm, *N* = 123), as shown in Figure 4a. The difference was statistically significant (Mann–Whitney *U* test, *p* < .05). The pooled data were consistent with the data collected nightly. Table 1 shows that the average SVL of amplectant males was larger than that of solo non‐amplectant males for all 10 nights for which we located amplexed pairs. The SVL of males (mean SVL ± SD =50.74 ± 2.01 mm, *N* = 30) and females (mean SVL ± SD = 97.72 ± 3.52 mm, *N* = 30) in amplexed pairs was not significantly correlated (linear‐regression analysis, *R* ^2^ = 0.000, *p* = 1.000, Figure 5). Thus, *O. graminea* showed no size‐assortative mating.

### 3.2 Correlation between male body size and call characteristics

**FIGURE 6:**
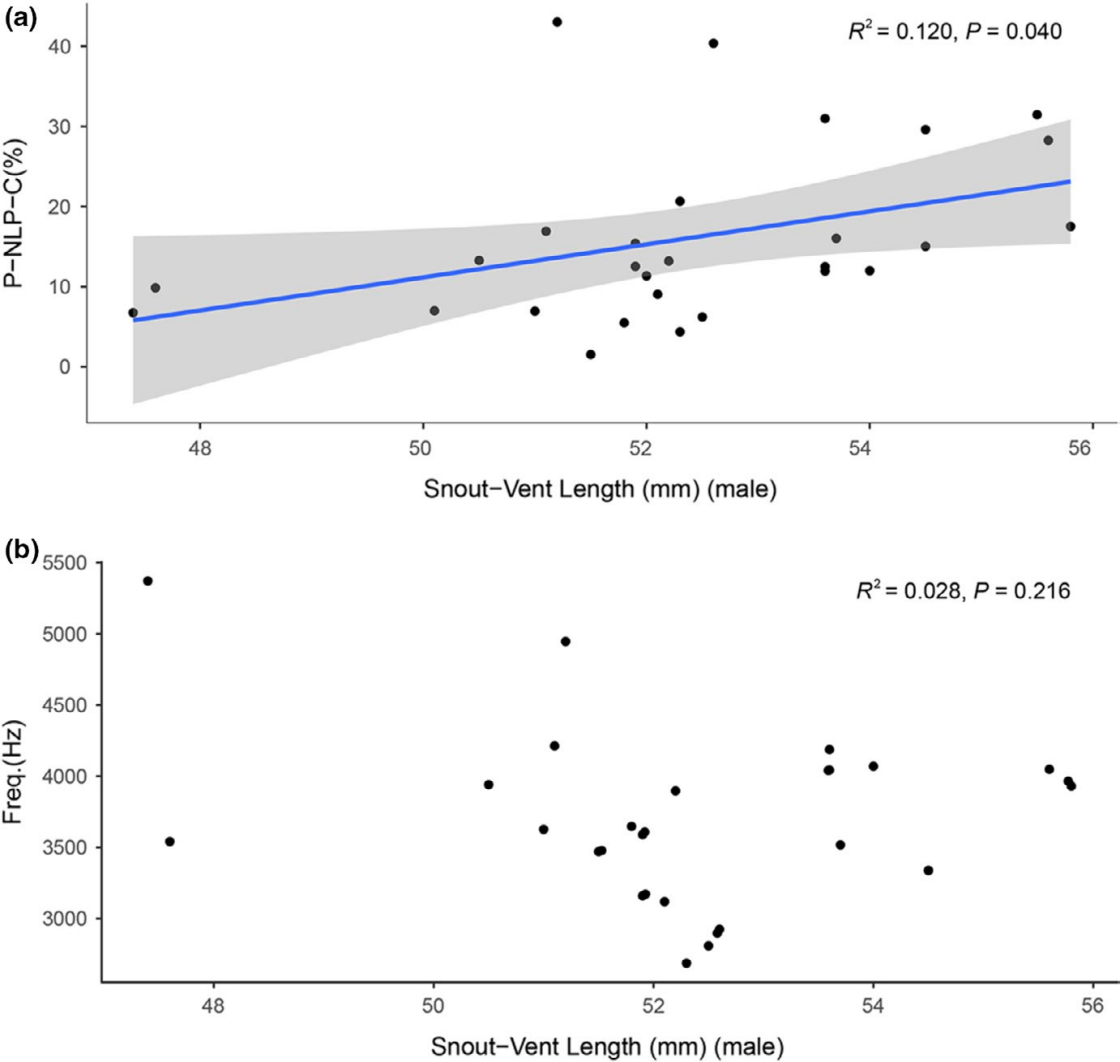
Correlation of the snout‐vent length (SVL) with the average P‐NLP‐C of male frogs (a) (*N* = 28, linear‐regression analysis, *p* < .05) and the fundamental frequency (b) (*N* = 28, linear‐regression analysis, *p* > .05). The gray areas represent the 95% confidence intervals

Analysis of the advertisement calls of 28 male frogs revealed that body size was significantly positively related to the P‐NLP‐C (linear‐regression analysis, *R* ^2^ = 0.120, *p* = .040, Figure 6a), but not significantly correlated with the fundamental frequency (linear‐regression analysis, *R* ^2^ = 0.028, *p* = .216, Figure 6b); in other words, calls of larger males showed a higher P‐NLP‐C, and P‐NLP‐C might be a potential predictor of male body size.

### 3.3 Female mate choice in amplexus experiments

In Experiment A, 30 females were subjected to the amplexus experiment; 3 females did not react, and 4 females chose smaller males; accordingly, 23 females indicated a preference for larger males. Based on all the video recordings, we confirmed the general pattern of *O. graminea* mate choice. After acclimating to the test terrarium, first, both males would gradually move closer to the female, accompanied by intermittent calls; then, the female would listen carefully and observe the two male competitors for a few minutes. After much deliberation, she would adjust her body direction and turn her back toward the favorite male. Finally, this male would call again in response to the female's choice, then jumped on the female's back to complete the amplexus. The mean SVL of the amplectant males was 50.53 mm (±1.64 SD, *N* = 27)—this differed significantly (paired *t* test, *p* < .001) from that of non‐amplectant males (mean ± SD = 48.41 ± 1.82 mm, *N* = 27) (Figure 4b). In Experiment B, we placed a new female with two males that had been previously paired with a different female. The results showed that larger males were once again chosen by all females (*N* = 20, 100%).

### 3.4 Female phonotaxis

**FIGURE 7:**
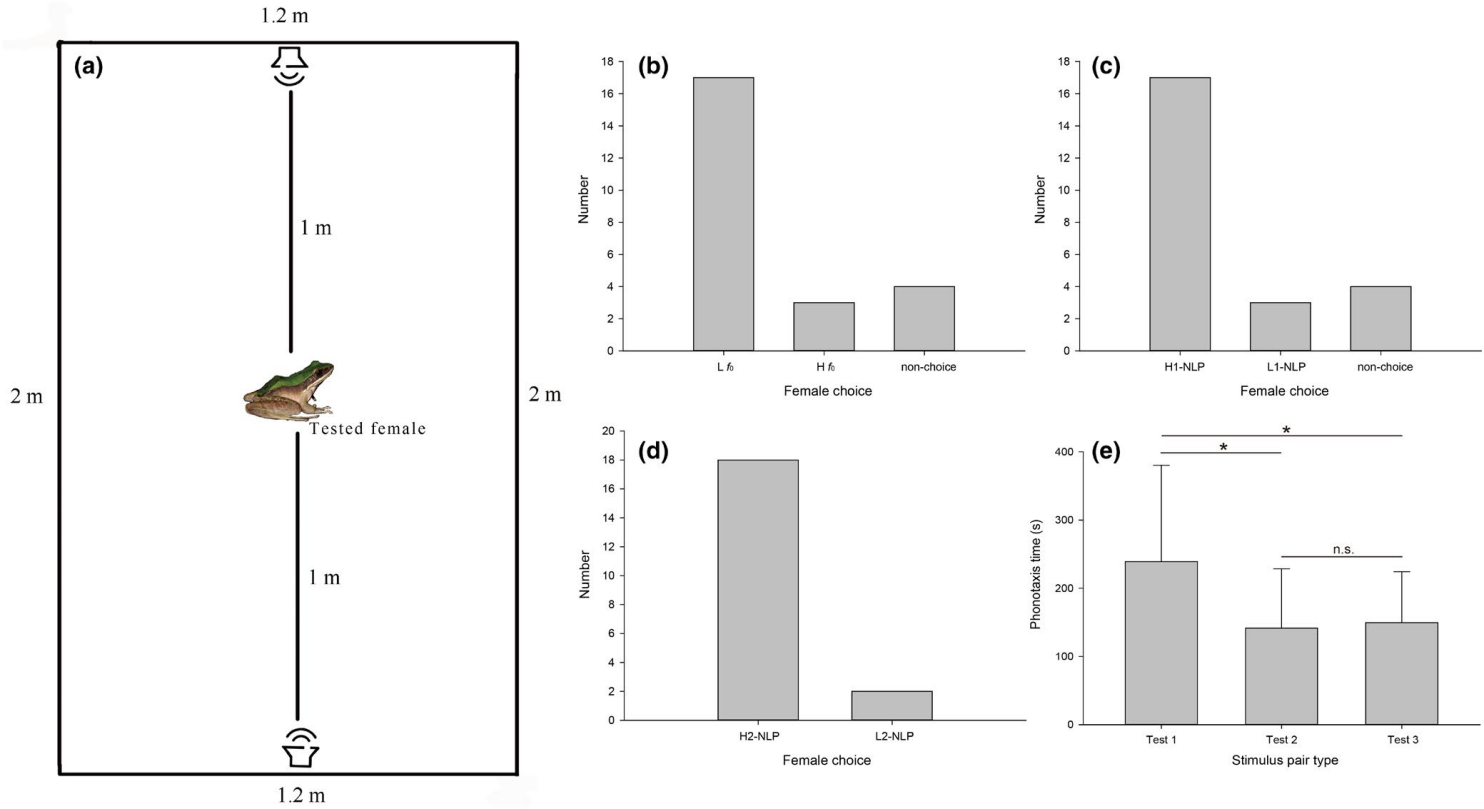
The results of female frog phonotaxis experiments. (a) Demonstration of experimental operation. (b) Female choice of test 1 (*N* = 24). (c) Female choice of test 2 (*N* = 24). (d) Female choice of test 3 (*N* = 20). (e) Difference of female phonotaxis times among different stimulus pairs (*N* = 20, Mann–Whitney U test, *p < .05; n.s. not statistically significant)

A total of 24 gravid female *O. graminea* were subjected to the phonotaxis experiments, and some females did not make a choice (*N* = 4); however, most female frogs preferred advertisement calls with a low *f*_0_ over calls with a high *f*_0_ (binomial test, *p* = .003, Figure 7b). Female frogs preferred advertisement calls with a high P‐NLP‐C (50%) compared to the advertisement calls with a low P‐NLP‐C (10%) (binomial test, *p* = .003, Figure 7c). Twenty gravid female frogs that responded in phonotaxis test 2 were subjected to phonotaxis test 3. The results of the experiment showed that calls with a high P‐NLP‐C (75%) were chosen by 18 females, while 2 females chose calls with a low P‐NLP‐C (20%) (binomial test, *p* < .001, Figure 7d).

Furthermore, Mann–Whitney *U* tests revealed that female phonotaxis times among different stimulus pairs were significantly different (Figure 7e). The female phonotaxis time was significantly longer for the stimulus pairs of L *f*_0_ vs H *f*_0_ (mean ± SD = 238.95 ± 141.38 s) compared to that of L1‐NLP vs H1‐NLP (mean ± SD = 141.50 ± 87.02 s) (Mann–Whitney *U* test, *p* < .05) and that of L2‐NLP vs H2‐NLP (mean ±SD =149.40 ± 74.90 s) (Mann–Whitney *U* test, *p* < .05). However, there was no significant difference in female phonotaxis time between test 2 and test 3 (Mann–Whitney *U* test, *p* = .66).

## 4 DISCUSSION

The results of our study in *O. graminea* support the working hypothesis that females would prefer males producing calls with a higher P‐NLP‐C. We found that in the field, females of *O. graminea* preferentially mated with larger males whose calls had a higher P‐NLP‐C. In manipulative experiments, females also showed a preference for males with larger body sizes and calls with a high P‐NLP‐C. The present study highlights the important role of NLP in anuran mate selection.

Anuran mating patterns are often nonrandom in terms of body size (Mansouri et al., 2020), which was also demonstrated in this study. Females of *O. graminea* preferentially mate with larger males in the field, which was validated in two two‐choice experiments. For most anuran species, females show a preference for larger males (Andersson, 1994; Rausch et al., 2014). Mating with larger males could result in indirect genetic benefits in terms of high offspring fitness. Márquez reported that midwife toads (*Alytes obstetricans* and *A. cisternasii*) who mate with larger males produce offspring with faster growth, a larger adult size, and superior survival ability (Márquez, 1995). But there are a few exceptions, for example, female serrate‐legged small treefrogs (*Philautus odontotarsus*) favored the intermediate size of males to reduce energy consumption for carrying male (Zhu et al., 2016). In addition, the body size ratio of amplexed pairs could affect the fertilization rate, for example, effective fertilization requires that cloaca to be properly juxtaposed during amplexus (Bastos & Haddad, 1996; Friedl & Klump, 2005). In *O. graminea*, there is greater female‐biased sexual size dimorphism between sexes (sexual dimorphism index = 1.93, *N* = 30; Figure S1) compared to most anuran species (Monnet & Cherry, 2002; Zhang & Lu, 2013); thus, choosing a large mate is conducive to improving the efficiency of amplexus and fertilization in *O. graminea*.

Due to individual differences in vocal apparatus, body size variation can affect the occurrence or intensity of NLP components in acoustic signals and increase the complexity of vocalizations (Cazau et al., 2016; Serrano et al., 2020). In *O. graminea*, a larger body size was associated with a higher P‐NLP‐C. The phonotaxis experiments indicated that the females preferred male calls with a higher P‐NLP‐C, so it is reasonable to speculate that a higher P‐NLP‐C in calls might enhance male attractiveness, affecting female mate choice and contributing to female preference for large males. It is likely that call features such as nonlinearities encode relevant information, such as body size (Juola & Searcy, 2011; Wu et al., 2021). The relationship between body size and NLP has been previously reported to occur in mammals (Cazau et al., 2016; Fitch et al., 2002), but it has not been corroborated by a significant correlation in any living organism. Serrano et al. (2020) first revealed that body size had influences on NLP components at the intra‐ and interpopulation levels, for example, in Darwin´s frogs (*Rhinoderma darwinii*), smaller individuals had higher proportions of relative duration of chaos. This study demonstrated that there was a significantly positive correlation between the P‐NLP‐C in calls and body size in male *O. graminea*. An inverse relationship between body size and fundamental frequency occurs typically in many anurans, but this study did not find the significant correlation in male *O. graminea*. It may be limited to the sample size of our study and require further experimental verification. Meanwhile, the evolution of female preference for male calls with different frequencies needs to take into account the multiple effects of auditory sensitivity and background context (Zhao et al., 2017). We speculate that males of *O. graminea* with bigger body size may not always have the calls with lower *f*_0_ in the field, otherwise, their opportunities of being detected by females would be reduced due to the masking of background stream noise. This suggests that for female *O. graminea*, the P‐NLP‐C may be a more reliable clue of male body size than the fundamental frequency. Further, this study also revealed that a higher P‐NLP‐C in courtship calls can enhance female attention to calling males, and larger males with a higher P‐NLP‐C had greater mating success than smaller rivals.

The complex structure and irregular frequency spectrum of NLP can increase the specificity of calls, making it easy to attract the attention of receivers and prevent habituation (Blumstein & Recapet, 2009). The results of phonotaxis experiments indicated that females of *O. graminea* had a strong preference for the calls with a high P‐NLP‐C or a low *f*_0_. However, the time of phonotaxis in the high‐low P‐NLP‐C tests was significantly less than that in the high‐low *f*_0_ test. This suggests that a high P‐NLP‐C might play a more important role in female phonotactic behavior than a low *f*_0_, and a higher P‐NLP‐C in calls has stronger effect on mate choice by females. These results are in accordance with recent findings in mammals, in which females displayed the strongest preference for calls with NLP over those without NLP (Charlton et al., 2017, 2018; Reby & Charlton, 2012). Therefore, the hypothesis that calls with nonlinear components are more attractive to females is not limited to mammals, as it also applies to other animals, including frogs.

The communicative significance of NLP has been examined in anurans (Feng, Arch, et al., 2009), reptiles (Labra et al., 2013), birds (Digby et al., 2014), and mammals (Tyson et al., 2007). Different functions have been assigned to NLP, including individual recognition (Feng, Riede, et al., 2009; Fitch et al., 2002), prevention of habituation (Karp et al., 2014), indicators of fitness (Fitch et al., 2002; Riede et al., 2004), and so on. However, the role of NLP in social interactions has rarely been evaluated (Digby et al., 2014). The study in *O. tormota* showed that male frogs with a high P‐NLP‐C in calls had a greater advantage in mate selection (Wu et al., 2021), consistent with the results of this study, indicating that the P‐NLP‐C in calls might play an important role in mate selection in frogs that can produce calls with NLP.

## CONFLICT OF INTEREST

The authors declare that they have no conflicts of interest.

## AUTHOR CONTRIBUTION

**Pan Chen:** Data curation (equal); Formal analysis (equal); Investigation (equal); Methodology (equal); Visualization (equal); Writing – original draft (equal); Writing – review & editing (equal). **Jinmei Wang:** Data curation (equal); Formal analysis (equal); Investigation (equal); Methodology (equal); Visualization (equal); Writing – original draft (equal). **Junqi Miao:** Formal analysis (equal); Investigation (supporting); Methodology (supporting); Visualization (supporting). **Hao Dong:** Formal analysis (supporting); Investigation (equal). **Jiahui Bao:** Investigation (supporting); Methodology (supporting). **Yatao Wu:** Formal analysis (supporting); Investigation (supporting); Methodology (supporting). **Fang Zhang:** Conceptualization (lead); Funding acquisition (lead); Methodology (lead); Project administration (lead); Resources (lead); Supervision (lead); Writing – review & editing (lead).

## Supporting information

Figure S1

## Data Availability

The raw data used to perform analyses and generate figures for this manuscript are available at https://doi.org/10.5061/dryad.sqv9s4n5h; https://doi.org/10.17632/xtrf2dxv6j.1.
